# Efficacy and Safety of Cerebrolysin for Acute Ischemic Stroke: A Meta-Analysis of Randomized Controlled Trials

**DOI:** 10.1155/2017/4191670

**Published:** 2017-06-05

**Authors:** Danfeng Zhang, Yan Dong, Ya Li, Jigang Chen, Junyu Wang, Lijun Hou

**Affiliations:** Department of Neurosurgery, Shanghai Institute of Neurosurgery, PLA Institute of Neurosurgery, Changzheng Hospital, Second Military Medical University, Shanghai, China

## Abstract

Cerebrolysin was reported to be effective in the neurological improvement of patients with acute ischemic stroke (AIS) in experimental models, while data from clinical trials were inconsistent. We performed a meta-analysis to explore the efficacy and safety of cerebrolysin for AIS. PubMed, EMBASE, and Cochrane Library were searched for randomized controlled trials, which intervened within 72 hours after the stroke onset. We investigated the efficacy and safety outcomes, respectively. Risk ratios and mean differences were pooled with fixed-effects model or random-effects model. Seven studies were identified, involving 1779 patients with AIS. The summary results failed to demonstrate significant superiority of cerebrolysin in the assessment of efficacy outcomes of mRS and BI. Similarly, administration of cerebrolysin had neutral effects on safety outcomes compared with placebo, including mortality and SAE. However, the number of included studies was small, especially in the analysis of efficacy outcomes, which might cause publication bias and inaccurate between-studies variance in the meta-analysis. Conclusively, although it seemed to be safe, routine use of cerebrolysin to improve the long-term rehabilitation after stroke could not be supported by available evidence.

## 1. Introduction

Acute ischemic stroke (AIS) is the second leading cause of death and one of the most common causes of adult disability worldwide [1, 2]. Thrombolytic therapy within 4.5 hours after the stroke onset can significantly reduce mortality and morbidity [3]. However, the effect is negligible after 4.5–6 hours [4] and useful for only a small portion of patients [5, 6]. The efficacy of aspirin and heparin was examined in two clinical studies, and only minimal benefit was detected for aspirin [7, 8].

In view of the unsatisfactory effects of current therapeutic options, neuroprotective therapies focusing on pathophysiological cascade and subsequent injuries were developed. Effects of compounds such as calcium antagonists, N-methyl-D-aspartate (NMDA) antagonists, anti-adhesion antibodies, and free oxygen radical scavengers were assessed [9, 10]. Despite potential benefits presented in experimental models, the results from clinical studies were somewhat disappointing [11, 12]. Reasons for the missing links between experimental and clinical studies were considered as the use of inappropriate experimental models and questionable design of current clinical trials [13].

Cerebrolysin is a neuropeptide preparation, consisting of low molecular weight neuropeptides and free amino acids. It has been suggested that cerebrolysin has neuroprotective and neurotrophic effects in cellular [14, 15], organic [16], and animal [17–19] AIS models through protecting neuron, inhibiting apoptosis, and reducing infarct size. However, conclusions were inconsistent in clinical trials [20–24]. Several studies found that cerebrolysin was helpful in bettering neurological outcomes and cerebral blood flow of AIS patients [20–23]. Another study including 1070 patients with AIS, however, demonstrated no benefits for cerebrolysin [24].

Therefore, we performed a systematic review and meta-analysis of randomized controlled trials (RCTs) to explore the efficacy and safety outcomes of cerebrolysin in patients with AIS.

## 2. Materials and Methods

### 2.1. Search Strategy

Our study was conducted following the recommendations of Preferred Reporting Items for Systematic Reviews and Meta-Analysis: The PRISMA Statement [25]. We intended to include RCTs comparing cerebrolysin and placebo in the treatment of AIS. Two authors (Danfeng Zhang and Yan Dong) searched PubMed, EMBASE, and Cochrane Library in July 2016 independently without date limits. The language was limited to English. We used terms cerebrolysin and stroke for the literature retrieval. References of included studies were examined for pertinent articles.

### 2.2. Selection Criteria

We included the studies if (1) they were clinical RCTs, (2) the subjects were adult with both genders, (3) they examined the efficacy and safety of cerebrolysin in the treatment of AIS, and (4) interventions were administrated within 72 hours after the stroke onset for at least 10 days.

### 2.3. Data Extraction and Assessment of Risk of Bias

Three investigators (Danfeng Zhang, Yan Dong, and Ya Li) extracted data on author name, publication year, study design, region, gender ratio, sample size, average age, time window of intervention, the efficacy outcomes, and safety outcomes at endpoint. Two reviewers (Lijun Hou and Junyu Wang) independently assessed the risk of bias of included studies according to the recommendation of Cochrane Collaboration, involving selection bias, performance bias, detection bias, attrition bias, and reporting bias [26]. The number of patients lost to follow-up was acceptable if it was less than 10% of the tested ensemble. We resorted to joint review if there were discrepancies among investigators.

### 2.4. Outcomes

Efficacy outcomes involved the evaluation of Barthel Index (BI) and modified Rankin Scale (mRS) at endpoint. Safety outcomes were mortality, adverse effect (AE), and severe adverse effect (SAE).

### 2.5. Statistical Analysis

Risk ratios (RRs) and mean differences (MDs) were selected as the effect sizes. Means and standard deviations (SDs) were calculated with Microsoft Office Excel 2007 (Microsoft Corporation, Washington) if the distribution of participants was available for quantitative variables. Heterogeneity was assessed with *I*^2^ statistic [27]. *I*^2^ values for low, moderate, and high heterogeneity were defined to be 25%, 50%, and 75% [27]. Fixed-effects model was used when *I*^2^ was less than 50% and *p* for heterogeneity exceeded 0.1. Otherwise, we used the random-effects model. We performed subgroup analyses according to the time window of intervention and sample size. For the sensitivity analysis, one study was excluded at a time. We also assessed the publication bias by Egger's test [28]. *p* < 0.1 was considered to be statistically significant in the test for the heterogeneity and publication bias [26, 28]. For other analyses, a significance level of *p* = 0.05 was used. Risk of bias was evaluated using Review Manager (RevMan), Version 5.3 (Copenhagen: the Nordic Cochrane Centre, the Cochrane Collaboration, 2014). Stata release 12 (StataCorp, College Station, TX) was used for the meta-analysis.

## 3. Result

### 3.1. Search Results

**Figure 1:**
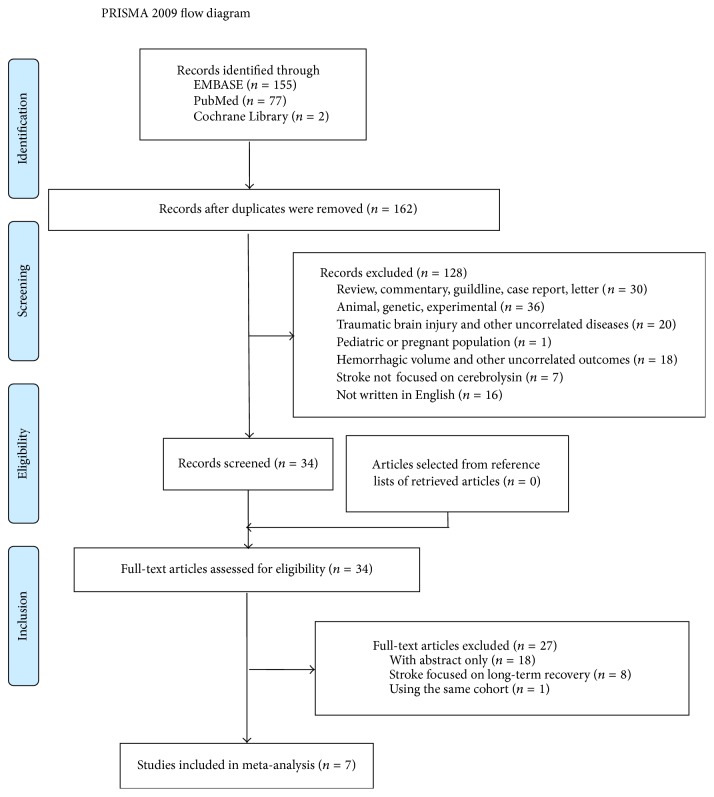
The flow diagram of the search process.

Detailed search process was shown in Figure 1. The initial retrieval produced 77 studies from PubMed, 155 studies from EMBASE, and 2 studies from Cochrane Library. 34 studies were left after the removal of duplicates and irrelevant studies. In the review of full text, seven studies were identified after excluding studies with incomplete data. No additional study was found in the review of references of these studies.

### 3.2. Study Characteristics and Risk of Bias

Table 1 demonstrated the main characteristics of 7 included RCTs [20–24, 29, 30], which involved 1779 AIS patients randomized within 72 hours after the stroke onset. Among them, 6 studies were published after 2010 [20–24, 30]. The age range was 18–85 and 61.2% of the subjects were males across studies. The follow-up duration of 6 studies was 90 days [20–22, 24, 29, 30], while it was 21 days in another study [23]. The summary risk of bias was presented in Figures S1 and S2 in Supplementary Material available online at https://doi.org/10.1155/2017/4191670. Intention-to-treat analyses were reported in seven studies [20–24, 29, 30]. Detailed data of efficacy outcomes and safety outcomes in included studies were available in Table S1 in Supplementary Material.

### 3.3. Efficacy Outcomes

#### 3.3.1. mRS

**Figure 2:**
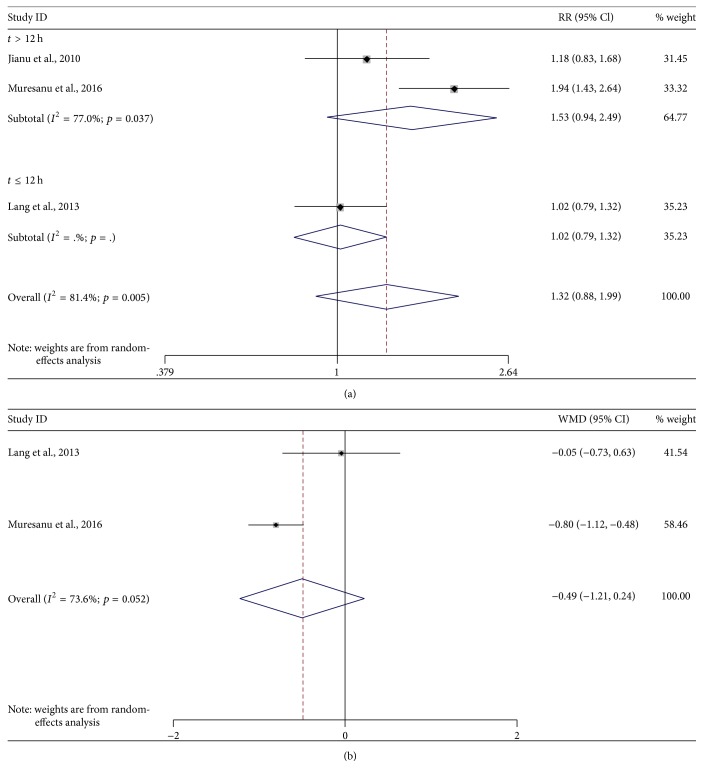
*Forest plots of cerebrolysin administration and mRS at endpoint*. (a) Subgroup analysis of dichotomous data for mRS defined by time widow of intervention. (b) Overall analysis of continuous data for mRS. CI, confidence interval; RR, risk ratio; WMD, weighted mean difference; mRS, modified Rankin Scale.

Three studies [20, 22, 30] reported mRS as efficacy outcome. Two of them provided both continuous and dichotomous data [22, 30], and another one provided only dichotomous data [20]. In the analysis of dichotomous data, a score of mRS ≤ 2 was considered as favorable outcome. The pooled RR was 1.32 (95% CI, 0.88–1.99, *p* = 0.18, Figure 2(a)) with evidence of high heterogeneity (*I*^2^ = 81%; *p* = 0.005) and random-effects model. In the analysis of continuous data, the summary MD was −0.49 (95% CI, −1.21–0.24, *p* = 0.19, Figure 2(b)) with moderate heterogeneity (*I*^2^ = 74%; *p* = 0.05) and random-effects model. Subgroup analysis for dichotomous data defined by time window of intervention (*p* = 0.895 for *t* ≤ 12 h and *p* = 0.089 for *t* > 12 h, Figure 2(a)) as well as the overall analysis for both data types indicated no statistically significant results.

#### 3.3.2. BI

**Figure 3:**
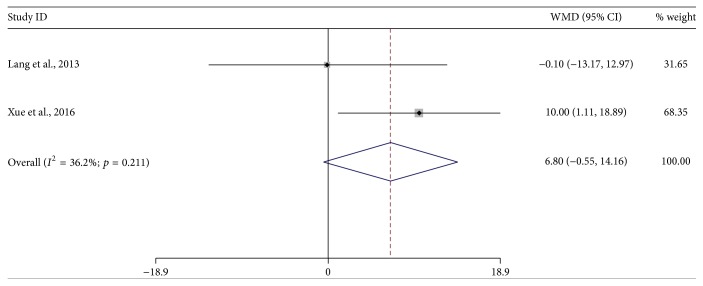
*Forest plots of cerebrolysin administration and BI at endpoint*. CI, confidence interval; WMD, weighted mean difference; BI, Barthel Index.

BI was included as efficacy outcome in two studies [23, 30] and expressed as continuous variable. The MD was 6.80 (95% CI, −0.55–14.16, *p* = 0.07, Figure 3) with low heterogeneity (*I*^2^ = 36%; *p* = 0.21) and fixed-effects model, demonstrating no statistically significant relationship between cerebrolysin and BI.

### 3.4. Safety Outcomes

#### 3.4.1. Mortality

**Figure 4:**
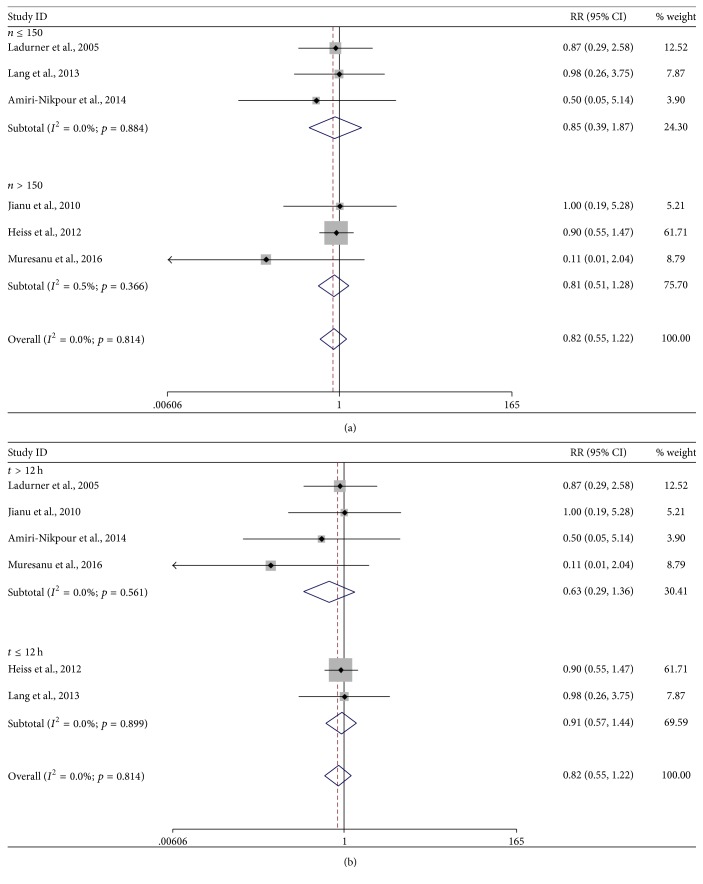
*Forest plots of cerebrolysin administration and mortality at endpoint*. (a) Subgroup analysis defined by sample size. (b) Subgroup analysis defined by the time widow of intervention. CI, confidence interval; RR, risk ratio.

Six studies were identified in the analysis of mortality [20–22, 24, 29, 30]. The overall meta-analysis suggested no significant difference in mortality between cerebrolysin and placebo with pooled RR of 0.82 (95% CI, 0.55–1.22, *p* = 0.33, Figure 4) and no heterogeneity (*I*^2^ = 0; *p* = 0.81). Subgroup analysis according to sample size and time window of intervention suggested no statistically significant results (*p* = 0.68 for *n* ≤ 150 and *p* = 0.37 for *n* > 150, Figure 4(a); *p* = 0.68 for *t* ≤ 12 h and *p* = 0.24 for *t* > 12 h, Figure 4(b)). There was no change when excluding studies one by one in sensitivity analysis. No publication bias was detected with Egger's test (*p* = 0.20).

#### 3.4.2. AE

**Figure 5:**
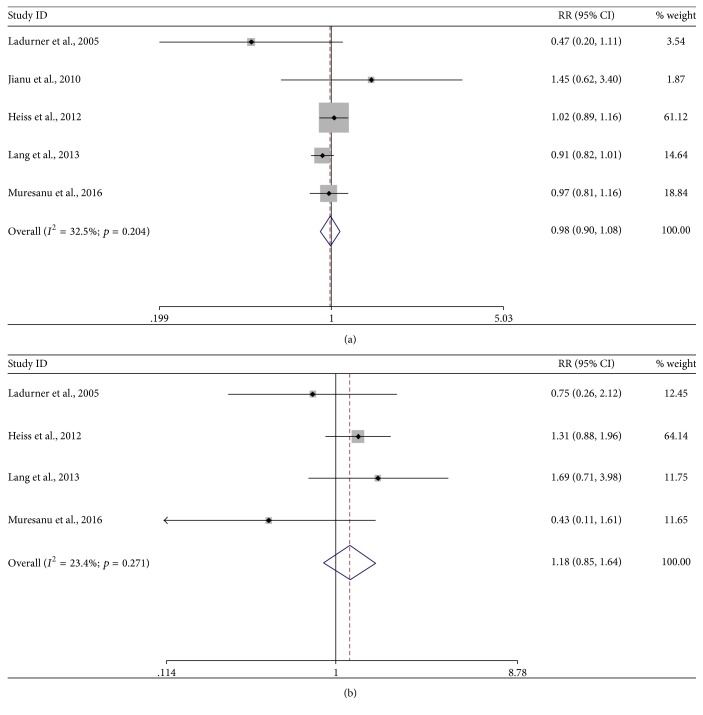
*Forest plots of cerebrolysin administration and AE and SAE at endpoint*. (a) Overall analysis for AE. (b) Overall analysis for SAE. CI, confidence interval; RR, risk ratio; AE, adverse event; SAE, serious adverse event.

Five studies assessed the effect of cerebrolysin on AE [20, 22, 24, 29, 30]. The overall analysis demonstrated no significant difference between cerebrolysin and placebo (RR, 0.98, 95% CI, 0.90–1.08, *p* = 0.75, Figure 5(a)) with low heterogeneity (*I*^2^ = 33%; *p* = 0.20) and fixed-effects model. In sensitivity analysis, no change was detected when removing studies one by one. There was no publication bias by Egger's test (*p* = 0.88).

#### 3.4.3. SAE

Four studies were available in the analysis of SAE [22, 24, 29, 30]. The summary RR failed to prove a significant difference between cerebrolysin and placebo (1.18, 95% CI, 0.85–1.64, *p* = 0.31, Figure 5(b)) with low heterogeneity (*I*^2^ = 23%; *p* = 0.27) and fixed-effects model. Sensitivity analysis suggested no altered result when removing studies one by one. Although only four studies were included, there was no publication bias by Egger's test (*p* = 0.33).

## 4. Discussion

In view of the wide prevalence and poor prognosis of AIS, it is crucial to develop effective therapies to improve neurological and cognitive functions of patients with AIS [1, 2]. Cerebrolysin, a neuroprotective compound, was tested in several clinical studies with inconsistent conclusions. According to our findings, no statistically significant result was detected for cerebrolysin in the analysis of mRS, BI, and safety outcomes compared with placebo, indicating that cerebrolysin seemed to be safe but of little benefit to AIS patients.

In the subgroup analysis of mortality defined by sample size, the summary RRs were also not statistically significant. The time window of intervention was 72 hours after the stroke onset in our study, which was a wide window of opportunity compared with other clinical trials on neuroprotective and thrombolytic therapies [3, 24]. According to a clinical RCT, the best effect of neuroprotective drugs was presented only in the first few hours of stroke onset [29]. However, in another study using tissue culture models of brain ischemia, cerebrolysin was reported to be effective in protecting neuron even after 72 hours since the stroke onset [31]. The time window of 72 hours in our study was disputable in the assessment of efficacy outcomes. Therefore, we conducted a subgroup analysis of studies which intervened within 12 hours after stroke onset. In the subgroup analysis of mortality and mRS, the overall results failed to confirm a significant effect for trials with a time window within 12 hours, which might be confused by the limited number of included studies.

Available data were limited for the meta-analysis of National Institutes of Health Stroke Scale (NIHSS), though it was frequently used as an efficacy outcome in clinical trials. The overall effect of cerebrolysin on NIHSS was inconsistent among clinical studies. Cerebrolysin was found to be beneficial for the improvement of NIHSS in three studies [21–23], while neutral effect was found in two studies [24, 30].

Cerebrolysin was shown to be neuroprotective and neurotrophic in preclinical studies [14, 15, 17–19]. The neuroprotective effects were exerted through counteracting excitotoxicity, inhibiting calpain and free radical formation in animal models [17–19]. The neurotrophic effects were demonstrated in cell culture studies [14, 15] and animal models [18] by inhibiting neuroinflammation, depressing apoptosis, and enhancing neurogenesis. All these effects contributed to the wide use of cerebrolysin in neurodegenerative diseases like Alzheimer's Disease and vascular dementia. In experimental model of AIS, intravenous administration of cerebrolysin could decrease infarct volume and mortality rate [19, 32].

Clinically, the use of cerebrolysin in patients with AIS has long been under debate. Among these debates, two recent studies suggested a beneficial role of cerebrolysin on short-term neurological outcomes after stroke with significant lower NIHSS scores and higher ARAT scores compared with placebo [22, 23]. Another study reported lower pulsatility index in the right middle cerebral artery compared to placebo [21]. In contrast, in a large scale Phase IV clinical trial, CASTA, neutral results on 90-day NIHSS between cerebrolysin and placebo groups were detected [24]. Because of underestimation of the significance of cognitive improvement on long-term rehabilitation after stroke, little concern was paid to the assessment of cognitive functions in clinical studies of cerebrolysin [20, 29]. In our review, two studies evaluating the syndrome short test and language function recovery after stroke were identified with superior effects for cerebrolysin in comparison with placebo [20, 29].

A recent systematic review examining the role of cerebrolysin in AIS indicated no enough evidence to support the routine administration of cerebrolysin to patients with AIS, which was in line with our findings [33]. However, only one study involving 146 participants was included [29]. In comparison, our investigation had strengths in including more studies with large sample size, involving relatively newer trials with dependable examinations and enrolling diverse population all over the world. In addition, besides safety outcomes, we assessed the efficacy outcomes of patients with AIS like mRS and BI.

However, several potential limitations are still in order. Firstly, different biases such as selection bias and publication bias do exist as a result of defects in study design of included trials and meta-analysis itself. Studies with positive results tend to be published. Moreover, we restricted the language of included studies to English and thus might overlook pertinent articles written in non-English languages. Secondly, the number of included studies was small, especially in the analysis of efficacy outcomes, which might cause publication bias and inaccurate between-studies variance in the meta-analysis. Moreover, results were inconsistent among clinical trials. A neutral effect was detected in two of three studies reporting the effect of cerebrolysin on mRS [20, 30], while Muresanu et al. reported favorable effect of cerebrolysin [22]. As for BI, only two studies were available with different conclusions [23, 30]. So caution was needed when interpreting these results. Thirdly, the effect of cerebrolysin on AIS had to do with several factors, such as damage zone, severity of injury, age of patients, time window of intervention, comorbidities, and combined therapies, which might increase the heterogeneity between studies. Small superiority for cerebrolysin was suggested in patients with more severe AIS compared with placebo [21, 24]. Amiri-Nikpour et al. reported lower mean flow velocity of basilar artery for patients below 65 years of age compared to patients over 65 years of age [21]. As for the time window of intervention, results were inconsistent and we did not find any significant effect in the subgroup analysis defined by time window. Meanwhile, cerebrolysin was all administrated within 72 hours in included studies, so more studies were needed for exploration of the effect of delayed administration after 72 hours. Because of the limited data, we could not draw conclusions concerning the effect of these confounders on the benefit of cerebrolysin as well as the population of stroke patients who may benefit from cerebrolysin. Fourthly, different neurological variables were used in included studies, which presented an obstacle to the meta-analysis of efficacy outcomes. For instance, cognitive functions were evaluated in two trials with different variables, making the meta-analysis of cognitive functions impossible [20, 29]. Finally, the follow-up durations of the included studies were mostly 90 days after the stoke onset, which restricted the evaluation of long-term rehabilitation of patients with AIS.

Despite the limitations, this meta-analysis presented some clinical implications. Although it seemed to be safe, routine use of cerebrolysin to improve the long-term prognosis after stroke could not be backed by available evidence. Meanwhile, our results should never be considered as the opposition of clinical administration of cerebrolysin in AIS. We advocate more clinical studies to unravel the exact effects and mechanisms of cerebrolysin and if possible identify the crowd who will benefit most from cerebrolysin. Future clinical trials perhaps need thorough design regarding time window of intervention, severity of stroke, unified outcome measures, combined therapies, and sample size.

## Supplementary Material

S1 Fig: Risk of bias graph.
S2 Fig: Risk of bias summary.
S1 Table: Original data of efficacy and safety outcomes in included studies. AE, adverse event; ARAT, Action Research Arm Test; BI, Barthel Index; CNS, Canadian Neurological Scale; GCS, Glasgow Coma Scale; GOS, Glasgow Outcome Scale; IQR, interquartile range; MMSE, Mini-Mental State Examination; mRS, modified Rankin Scale; NIHSS, National Institutes of Health Stroke Scale; SAE, serious adverse event; SD, standard deviation; SST, syndrome short test; UNSS, Unified Neurological Stroke Scale. ∗Post-baseline mRS data were not a vailable for five patients in the cerebrolysin group. ^†^Data expressed as mean (95% CI); ^‡^Data expressed as median (IQR).

## Figures and Tables

**Table 1:** Characteristics of the included studies.

First author, publication year, country	Sample size (% men)	Population age (years)	Time window from stroke onset to intervention (hours)	Intervention	Control	Efficacy outcomes	Safety outcomes	Time window from stroke onset to examination (days)	Baseline NIHSS
Ladurner, 2005, Austria, Czech Republic, and Hungary	146 (58.2)	45–85	24	Cerebrolysin for 21 days, 50 mL/d, IV	Placebo for 21 days	CNS, BI, GCS, CGI, MMSE, SST, HAMD	AE, SAE, lab tests, vital signs	1, 3, 7, 14, 21, 90	NA
Jianu, 2010, Romania	156 (71.8)	20–75	72	Cerebrolysin for 21 days, 30 mL/d, IV	Placebo for 21 days	mRS, mortality	AE	90	14
Heiss, 2012, China, Hong Kong, Republic of Korea, and Myanmar	1067 (60)	18–85	12	Cerebrolysin for 10 days, 30 mL/d, IV, in addition to aspirin (100 mg/d)	Placebo for 10 days in addition to aspirin (100 mg/d)	mRS, BI, NIHSS	AE, SAE, lab tests, vital signs	90	9
Lang, 2013, Austria, Croatia, Czech Republic, Slovenia, and UK	119 (64.7)	18–80	3	Cerebrolysin for 10 days, 30 mL/d, IV	Placebo for 10 days	mRS, NIHSS, BI, GOS	AE, SAE, lab tests, vital signs	5, 10, 30, 90	Active: 12.3
									Control: 11
Amiri-Nikpour, 2014, Iran	46 (51.2)	18–85	12–30	Cerebrolysin for 10 days, 30 mL/d, IV, adjunct to 100 mg of aspirin daily	Placebo for 10 days adjunct to 100 mg of aspirin daily	NIHSS, mean flow velocity (cm/s) of cerebral arteries, PI	NA	30, 60, 90	14
Muresanu, 2016, Romania, Ukraine, and Poland	205 (63.9)	18–80	24–72	Cerebrolysin for 21 days, 30 mL/d, IV	Placebo for 21 days	ARAT, NIHSS, BI, mRS	AE, SAE, lab tests, vital signs	42, 90	Active: 9.1
									Control: 9.2
Xue, 2016, China	40 (47.5)	52–87	12	Cerebrolysin for 10 days, 30 mL/d, IV	Placebo for 10 days	NIHSS, BI	Adverse events, lab tests, vital signs	11 and 21 days after the initiation of therapy	Active: 10.6
									Control: 10.2

AE, adverse event; ARAT, Action Research Arm Test; BI, Barthel Index; CGI, Clinical Global Impressions; CNS, Canadian Neurological Scale; GCS, Glasgow Coma Scale; GOS, Glasgow Outcome Scale; HAMD, Hamilton Rating Scale for Depression; IQR, interquartile range; MMSE, Mini-Mental State Examination; mRS, modified Rankin Scale; NA, not available; NIHSS, National Institutes of Health Stroke Scale; PI, pulsatility index; SAE, serious adverse event; SD, standard deviation; SST, syndrome short test; UNSS, Unified Neurological Stroke Scale.
