# Supplementary material for: Efficacy and Safety of Cerebrolysin for Acute Ischemic Stroke: A Meta-Analysis of Randomized Controlled Trials
Source: Biomed Res Int. 2017 Jun 5;2017:4191670. doi: 10.1155/2017/4191670 (PMC5474547; doi:10.1155/2017/4191670)
Supplement: Supplementary file 1 — S1 Fig: Risk of bias graph. S2 Fig: Risk of bias summary. S1 Table: Original data of efficacy and safety outcomes in included studies. AE, adverse event; ARAT, Action Research Arm Test; BI, Barthel Index; CNS, Canadian Neurological Scale; GCS, Glasgow Coma Scale; GOS, Glasgow Outcome Scale; IQR, interquartile range; MMSE, Mini-Mental State Examination; mRS, modified Rankin Scale; NIHSS, National Institutes of Health Stroke Scale; SAE, serious adverse event; SD, standard deviation; SST, syndrome short test; UNSS, Unified Neurological Stroke Scale. ∗Post-baseline mRS data were not a vailable for five patients in the cerebrolysin group. †Data expressed as mean (95% CI); ‡Data expressed as median (IQR). [file 4191670.f1.pdf]

SUPPLEMENTARY MATERIALS

Efficacy and Safety of Cerebrolysin for Acute Ischemic Stroke: A Meta-analysis of Randomized Controlled Trials.

Danfeng Zhang, Yan Dong, Ya Li, Jigang Chen, Junyu Wang, Lijun Hou

S1 Fig. Risk of bias graph.

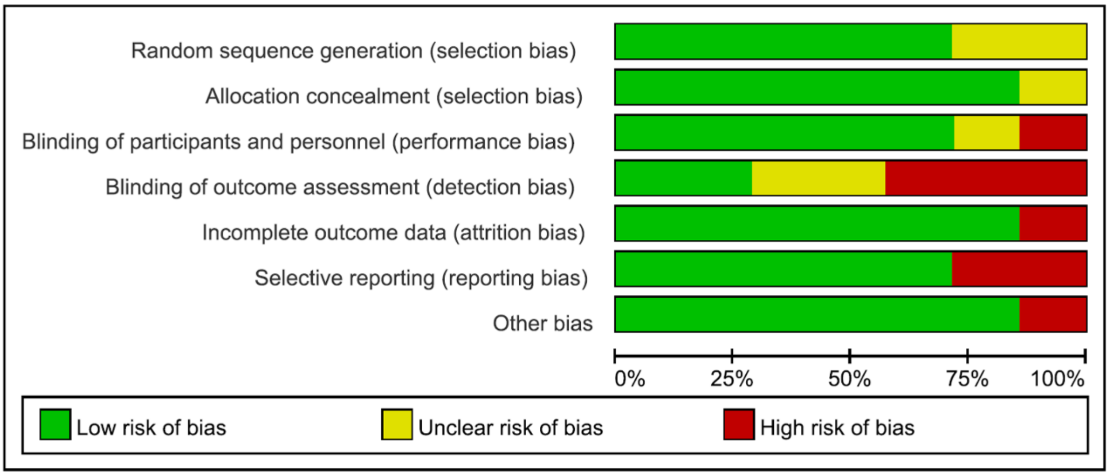

S2 Fig. Risk of bias summary.

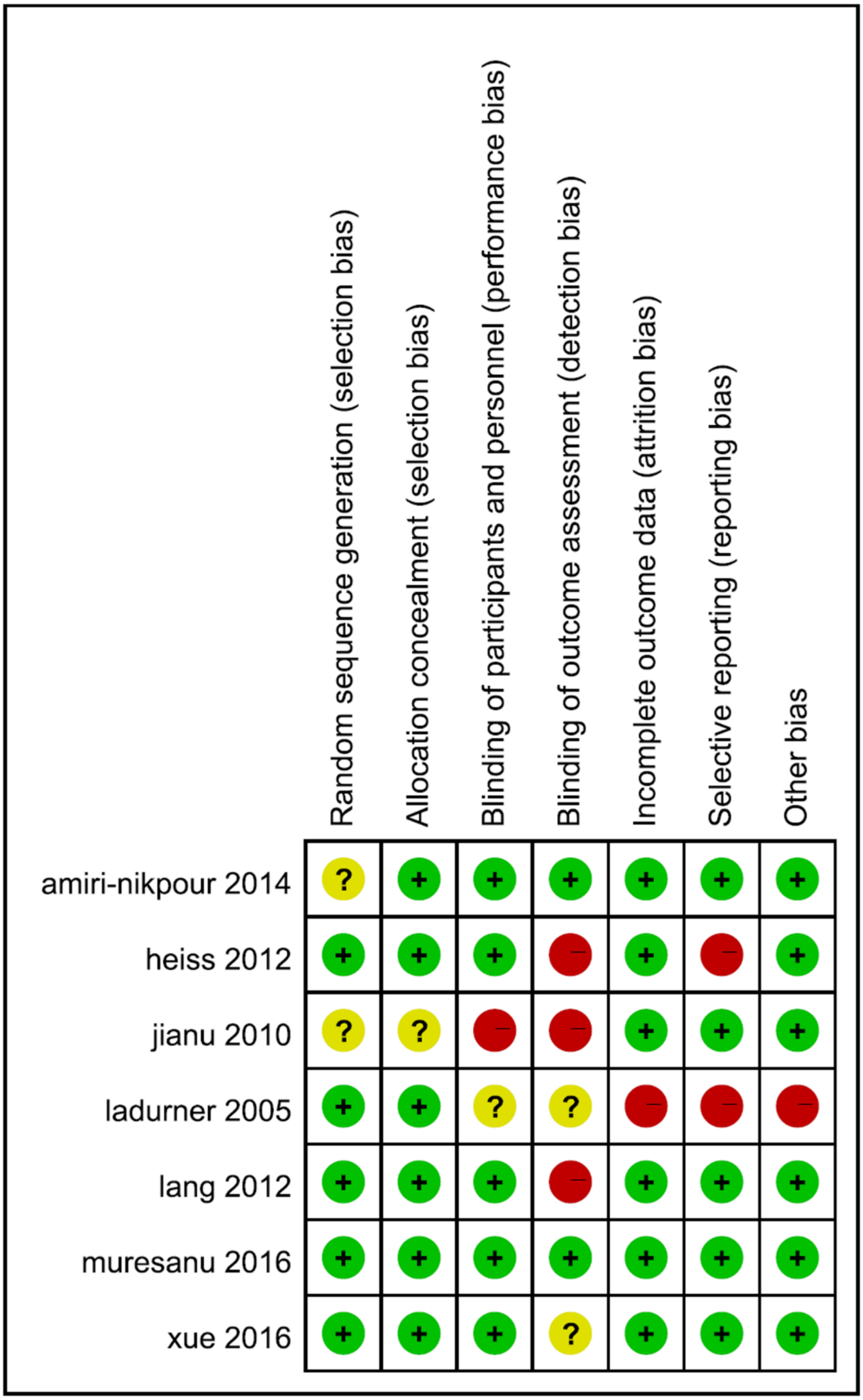

S1 Table. Original data of efficacy and safety outcomes in included studies.

Author name, publication year	Study objective	number of intervention group vs. placebo group	Efficacy and safety assessment	Number/(mean ±SD)/mean(95% CI)/median(IQR) of active treatment group vs. placebo group
Ladurner, 2005	acute ischemic stroke	78 vs. 68	AE	7 vs. 13
Ladurner, 2005	acute ischemic stroke	78 vs. 68	SAE	6 vs. 7
Ladurner, 2005	acute ischemic stroke	78 vs. 68	Death	6 vs. 6
Jianu, 2010	acute ischemic stroke	52 vs. 104	AE	8 vs. 11
Jianu, 2010	acute ischemic stroke	52 vs. 104	mRS	26 vs. 44
Jianu, 2010	acute ischemic stroke	52 vs. 104	Death	2 vs. 4
Heiss, 2012	acute ischemic stroke	527 vs. 540	AE	242 vs. 243
Heiss, 2012	acute ischemic stroke	527 vs. 540	SAE	50 vs. 39
Heiss, 2012	acute ischemic stroke	527 vs. 540	Death	28 vs. 32
Lang, 2012	acute ischemic stroke	60 vs. 59	mRS	37 vs. 39*
Lang, 2012	acute ischemic stroke	60 vs. 59	mRS	1.93 ±1.91 vs. 1.98 ±1.81
Lang, 2012	acute ischemic stroke	60 vs. 59	GOS	30 vs. 33
Lang, 2012	acute ischemic stroke	60 vs. 59	BI	76·57 (67·20, 85·94) vs. 76·67 (67·55, 85·78) †,
Lang, 2012	acute ischemic stroke	60 vs. 59	AE	53 vs. 57
Lang, 2012	acute ischemic stroke	60 vs. 59	SAE	12 vs. 7
Lang, 2012	acute ischemic stroke	60 vs. 59	Death	4 vs. 4
Amiri-Nikpour, 2014	acute ischemic stroke	23 vs. 23	NIHSS	9 (8, 10) vs. 11 (10, 13.5) ‡
Amiri-Nikpour, 2014	acute ischemic stroke	23 vs. 23	Death	1 vs. 2
Muresanu,	acute ischemic	104 vs. 101	ARAT	40.7±20.2 vs. 26.5±21.0

2016	stroke			
Muresanu, 2016	acute ischemic stroke	104 vs. 101	mRS	68 vs. 34
Muresanu, 2016	acute ischemic stroke	104 vs. 101	mRS	1.97 ± 1.22 vs. 2.77 ± 1.12
Muresanu, 2016	acute ischemic stroke	104 vs. 104	AE	72 vs. 74
Muresanu, 2016	acute ischemic stroke	104 vs. 104	SAE	3 vs. 7
Muresanu, 2016	acute ischemic stroke	104 vs. 104	Death	0 vs. 4
Xue, 2016	acute ischemic stroke	20 vs. 20	NIHSS	5.90±3.96 vs. 7.30±4.78
Xue, 2016	acute ischemic stroke	20 vs. 20	BI	53.75±13.10 vs. 43.75±15.50

AE, adverse event; ARAT, Action Research Arm Test; BI, Barthel Index; CNS, Canadian Neurological Scale; GCS, Glasgow Coma Scale; GOS, Glasgow Outcome Scale; IQR, interquartile range; MMSE, Mini-Mental State Examination; mRS, modified Rankin Scale; NIHSS, National Institutes of Health Stroke Scale; SAE, serious adverse event; SD, standard deviation; SST, syndrome short test; UNSS, Unified Neurological Stroke Scale.

*Post-baseline mRS data were not available for five patients in the cerebrolysin group.

†Data expressed as mean (95% CI); ‡ Data expressed as median (IQR).
